# Rapid Multi-Locus Sequence Typing Using Microfluidic Biochips

**DOI:** 10.1371/journal.pone.0010595

**Published:** 2010-05-12

**Authors:** Timothy D. Read, Rosemary S. Turingan, Christopher Cook, Heidi Giese, Ulrich Hans Thomann, Catherine C. Hogan, Eugene Tan, Richard F. Selden

**Affiliations:** 1 Biological Defense Research Directorate, Naval Medical Research Center, Rockville, Maryland, United States of America; 2 Division of Infectious Diseases, Department of Medicine, and Department of Human Genetics, Emory University School of Medicine, Atlanta, Georgia, United States of America; 3 Network Biosystems Inc, Woburn, Massachusetts, United States of America; University of Hyderabad, India

## Abstract

**Background:**

Multiple locus sequence typing (MLST) has become a central genotyping strategy for analysis of bacterial populations. The scheme involves *de novo* sequencing of 6–8 housekeeping loci to assign unique sequence types. In this work we adapted MLST to a rapid microfluidics platform in order to enhance speed and reduce laboratory labor time.

**Methodology/Principal Findings:**

Using two integrated microfluidic devices, DNA was purified from 100 *Bacillus cereus* soil isolates, used as a template for multiplex amplification of 7 loci and sequenced on forward and reverse strands. The time on instrument from loading genomic DNA to generation of electropherograms was only 1.5 hours. We obtained full-length sequence of all seven MLST alleles from 84 representing 46 different Sequence Types. At least one allele could be sequenced from a further 15 strains. The nucleotide diversity of *B. cereus* isolated in this study from one location in Rockville, Maryland (0.04 substitutions per site) was found to be as great as the global collection of isolates.

**Conclusions/Significance:**

Biogeographical investigation of pathogens is only one of a panoply of possible applications of microfluidics based MLST; others include microbiologic forensics, biothreat identification, and rapid characterization of human clinical samples.

## Introduction

Multiple locus sequence typing, developed in the early 1990s [1], has become a central genotyping strategy for analysis of bacterial populations. For a typical scheme, conserved oligonucleotide primers are designed to amplify 300–600 bp fragments of 6–8 housekeeping genes. Bacterial strains are assigned to unique combinations of alleles called Sequence Types (STs). MLST allows direct interrogation of nucleotide sequence variation, in contrast to fingerprinting approaches such as AFLP (amplified fragment length polymorphism), VNTR (variable number of tandem repeats) and RFLP (restriction fragment length polymorphism) [2]. Although whole genomic shotgun sequencing using ‘next-generation’ sequencing technologies [3] is orders of magnitude more efficient on a per-nucleotide basis, MLST is a more rapid and cost effective method for ascertaining the genetic structure of large strain collections, particularly when many of the isolates may be isogenic. The increasing popularity of MLST is reflected in the almost 50 species and thousands of individual isolates stored in two of the largest publicly accessible databases, www.mlst.net and www.pubmlst.org. MLST has been used in many studies to explore the evolution and demography of pathogenic bacteria [1], [4], [5], [6], [7].

Despite increased interest in the scientific approach, the laboratory methodology for producing MLST profiles is essentially unchanged in 20 years. For an individual bacterial strain, genomic DNA isolation, amplification of each locus and sequencing the forward and reverse strands are performed as separate reactions using different sets of instrumentation. A significant amount of labor and bookkeeping are required for genotyping a large strain collection, presenting an economic barrier to the generation of highly informative data sets. Moreover, the substantial investment in equipment, supplies and training currently limits the application of MLST for rapid screening of isolates in clinical microbiology laboratories. Here, we present data on a focused sequencing approach that employs microfluidic technologies to reduce the experimental complexity of MLST by running multiplex PCR, Sanger sequencing reactions, ultrafiltration, electrophoretic separation, and laser-induced fluorescence detection of sequenced fragments on microfluidic biochips. In this manuscript, we refer to the microfluidic MLST methodology as ‘mMLST’ to distinguish it from the traditional MLST approach.

## Results

### Development of an mMLST approach for *B. cereus*

**Figure 1:**
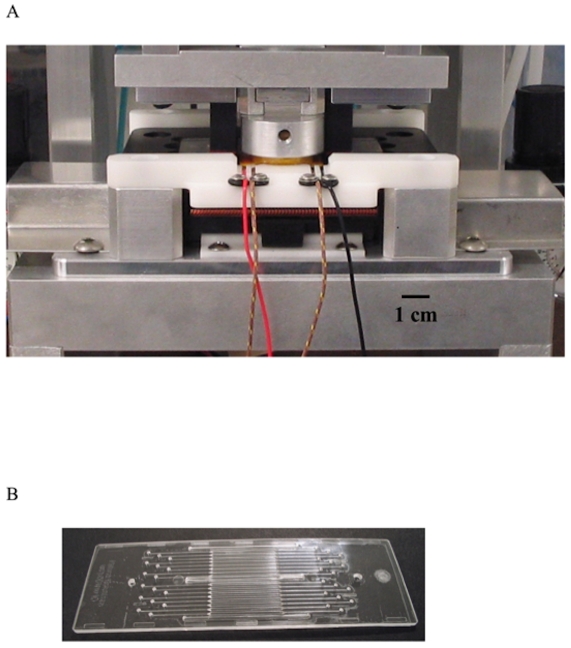
Microfluidic thermal cycling for PCR and Sanger sequencing. A) Rapid, Peltier-based thermal cycler; B) 16 sample microfluidic biochip (dimension 25×75 mm).

**Figure 2:**
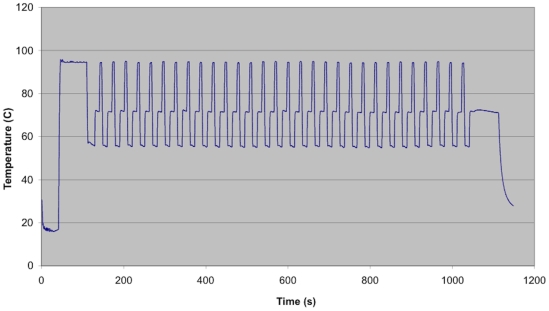
Rapid thermal cycler solution temperature profile utilized for rapid, multiplexed amplification.

Two instruments designed by Network Biosystems were used in the course of these experiments; a fast thermal cycler for PCR and sequencing and the Genebench-FX instrument for high resolution separation and detection. The thermal cycler (**Figure 1a**) consisted of three main subsystems: a heat pump providing a variable temperature-controlled surface on which the microfluidic biochip (**Figure 1b**) is positioned, a compression case that clamps the biochip to the heat pump, and electronic hardware and software that accurately control and maintain cycled temperature profile. The instrument was capable of reaction solution temperature ramp rates in excess of 15°C/sec with 0.5°C stability at the dwell temperatures (**Figure 2**). Power was supplied as forward or reverse DC current, modulated through a high power H-bridge amplifier. DNA for amplification was loaded onto an instrument-specific 25×75 mm biochip. The biochip has 16 independent reaction chambers with dimensions of 0.8 mm×15 mm×0.5 mm, holding approximately 6 µl each. Close contact between the instrument and biochip, essential for minimizing thermal response time, was achieved by pneumatic compression. The same biochip design was used for both PCR and Sanger thermal cycling, and each disposable biochip was utilized for a single reaction. Electrophoretic separation of DNA fragments that were the product of the Sanger sequencing reactions took place within a second microfluidic biochip on the Genebench-FX instrument prefilled with sieving matrix specifically designed and optimized for rapid, high resolution and high sensitivity separation performance. Loading of the DNA from the sample wells to the separation channel and separation of the DNA within the separation channel were controlled by on-board software. DNA from the sample wells was moved to the loading zone of the separation channel by the application of a voltage across the sample and waste reservoirs, generating a loading electric field that moved the DNA towards the separation. DNA fragments travelled down the separation channel until they arrived at the detection zone, where they were exposed to the laser. The dye molecules that were attached to the sequenced DNA fragments absorbed energy from the laser and emitted fluorescence in the visible spectrum. The dye-specific emitted fluorescence was collected by an optical train and transmitted to a color-specific detector. The run time for electrophoresis of up to 550 nt DNA fragments was about 25 minutes.

**Figure 3:**
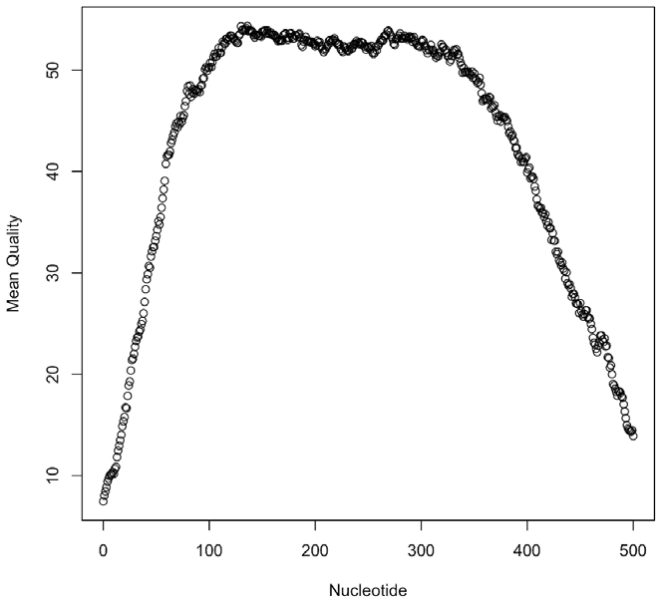
Average quality score over fragment length. The average quality score [10] for each position from the start of the untrimmed read was calculated for the *glp* locus using a custom perl script (**Text S1**). Data points represent the average of 198 electropherograms. The fall-off in quality between 400–500 nucleotides coincides with the average end of the amplicons. The *glp* locus data quality is similar to that of the other MLST loci (**Figure S4**). Means and standard deviations for all points are available in the **Data S3** file.

The organism chosen for this study was the *Bacillus cereus* sensu lato group (s. l; translation - ‘in the broad sense’), a polyphyletic species of Gram positive bacteria that includes *B. cereus*, *B. anthracis*, *B. thuringiensis*, *B. mycoides* and *B. weihenstephanensis* [8], [9]. The primer pairs used for PCR [6] amplified fragments of seven *B. cereus* s.l. housekeeping genes with sizes ranging from 348–504 base pairs. PCR amplification conditions were optimized for the microfluidic thermal cycler using *B. cereus* s.l. To validate the accuracy of the system, frozen cell pastes of 8 *B. cereus* s.l. group strains with known STs were supplied to the test laboratory in a blinded test. DNA was prepared by a simple guanidinium hydrochloride extraction (see methods). All PCR reactions and all Sanger sequencing runs on the microfluidic sequencer for all eight strains were successful. The quality scores [10] obtained with the Genebench-FX biochip using the nnimbc4 basecalling software matched results from ‘conventional’ Sanger capillary electrophoresis equipment (**Figure 3**). The base quality, or ‘Q’, score is defined as -10log_10_P, where P is the error probability. Therefore, Q scores of 20 and 40 refer to base error probabilities of 0.01 and 0.0001, respectively. The average number of bases of Q20 or higher in forward and reverse reads of each locus (with the total length of the amplicon in parentheses) were: *gmk*: 464 (504); *tpi*: 459 (435); *pycA*: 415 (363); *glpF*: 430 (381); *pta*: 453 (414); *ilvD*: 417 (393); *pur*: 427 (348).

The assembled sequences of all the alleles matched the expected sequence in the MLST database with 100% accuracy. As the concatenated length of the amplicons for each of the eight strains was 2,892 bp, the sequencing error was estimated at less than 1 base in 23,000. The average quality score of the assembled sequences was therefore >43, in line with average quality scores of >40 predicted by the basecalling software on the forward and reverse strands.

### mMLST typing of an environmental population of *Bacillus cereus* strains

**Figure 4:**
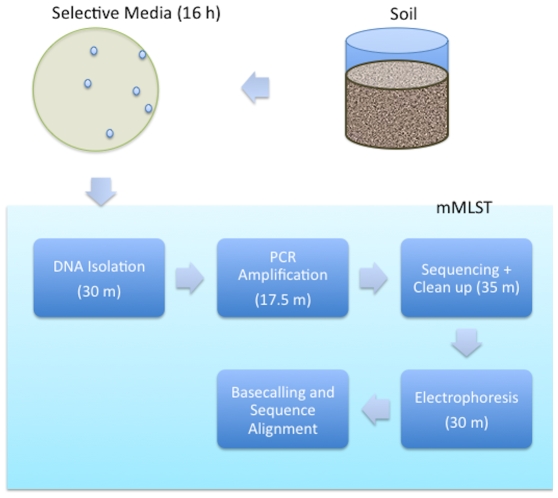
Workflow of mMLST sequencing.

To investigate how mMLST could be applied to studies of pathogen and near-neighbor populations, we sequenced a collection of 100 *B. cereus* s. l. primary environmental strains (**Figure 4**). The bacteria were isolated from soil shaded by trees in Rockville, Maryland using selective *B. cereus* agar [11]. All seven amplicons were generated by PCR and successfully sequenced for 84 strains (**Data S1**). For fifteen strains only a subset of the MLST loci could be amplified. The reasons for PCR failure in these strains were a combination of non-specificity of the *ilvD* locus primers in particular and nucleotide sequence divergence of the genome DNA templates in general. The *ilvD* locus has the highest nucleotide diversity of the seven used for MLST [6] and 9/15 incomplete strains failed PCR only at this locus. PCR was successful for one strain of the fifteen strains (S6570) using alternative primers for amplification (*ilvD-2* and *ilv4F*; www.pubmlst.org). A neighbor-joining tree of the *glp* locus (the primer pair that was successful in amplifying across the broadest range of isolates) showed that several of the strains missing alleles were deeply diverged from the three main *B. cereus* s.l. clades (**Figure S1**). The genetic diversity of bacteria isolated using *B. cereus* selective agar was therefore greater than can be completely sampled using the existing *B. cereus* MLST primer sets. However, only one strain out of 100 identified failed to amplify any allele. Sequencing of 16S ribosomal coding sequence revealed that this was in fact a *Bacillus megaterium* strain. The average Q scores and standard deviation at each position for all the sequenced loci are given in **Text S1**.

**Figure 5:**
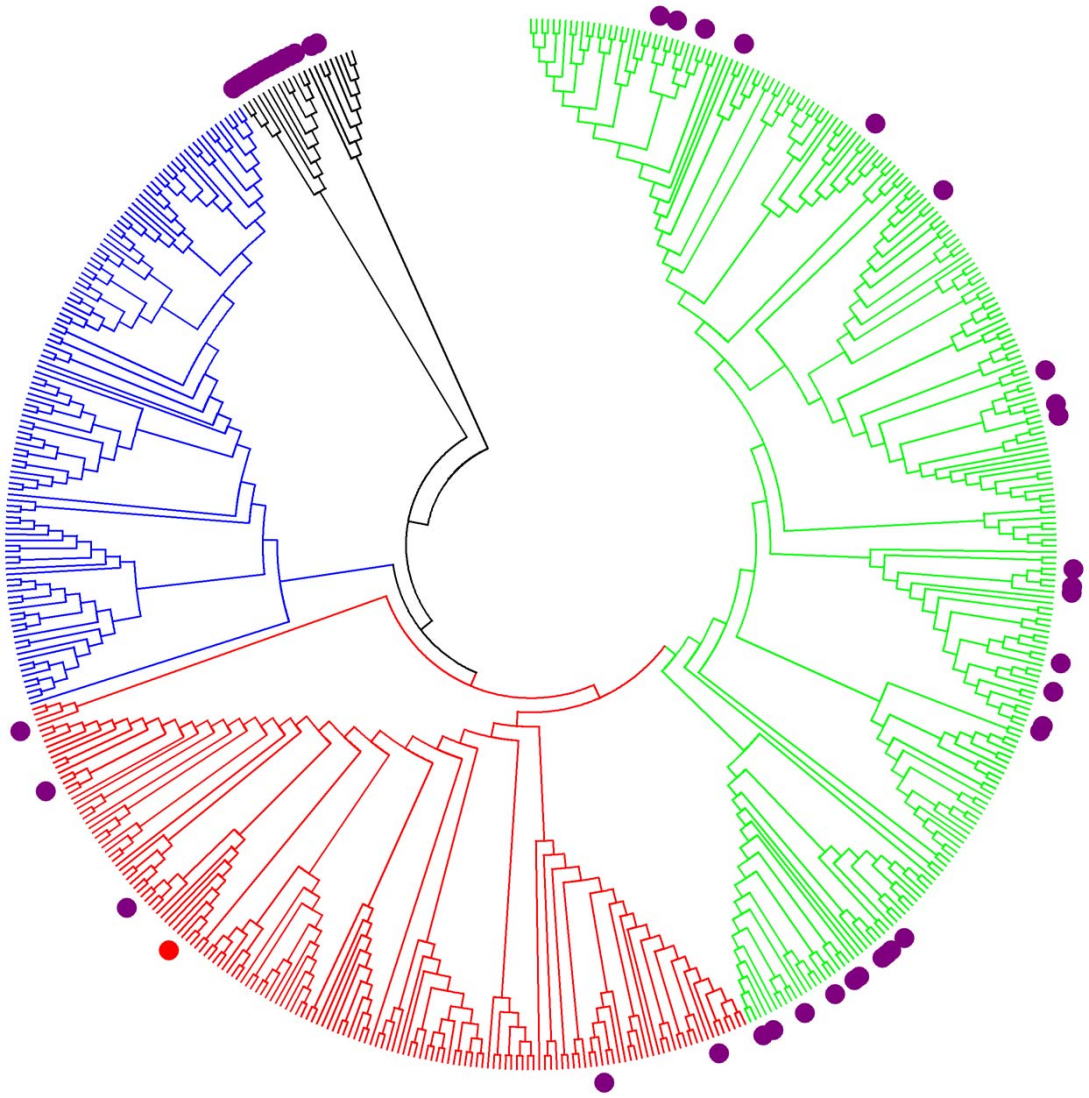
Phylogeny of *B. cereus* Sequence Types. The evolutionary history of all the *B. cereus* MLST concatenated Sequence Types (545 taxa, 2,394 nucleotide positions) was inferred using the Neighbor-Joining method [28]. The bootstrap consensus tree inferred from 100 replicates was taken to represent the evolutionary history of the taxa. Evolutionary Distances were computed using the Maximum Composite Likelihood method [15] and are in the units of number of base substitutions per site. The tree was rooted at ST83 (*B. pseudomycoides*) and clade designations were based on the presence of STs identified by Barker et al [29]. Clade 1 branches are shown in red, clade 2 in green, clade 3 in blue and outgroup strains in black. The Rockville STs are shown as purple circles. For reference, *B. anthracis* (ST1) is shown as a red circle.

The sequence data was uploaded into the pubmlst.org database in January 2009. 55 novel strains representing 37 new Sequence Types and 236 previously unreported allele sequences were assigned to the new *B. cereus* s.l. isolates. The 84 complete strains were distributed over 46 STs with a logarithmic ranked abundance curve (**Data S2**). The 84 complete Rockville strains were distributed across major branches of the *B. cereus* s.l. phylogeny, including a few that are members of diverged outgroups (**Figure 5**). The structure of the tree produced in figure 5 is broadly similar to that described in other analyses of the *B. cereus* MLST dataset [6], [12], [13]. The largest number of Rockville strains fell in clade 2 (49) with 30 strains falling in deeply branched outgroups (30). Interestingly, no Rockville isolates had STs that matched those attributed to clade 3 strains, which have been found in other studies to be the most prevalent in soils [11] [14]. Clade 3, containing mainly psychrophiles, is likely underrepresented in *B. cereus* s.l. strain collections [5], [6], while clade 2 contains many *B. thuringiensis* strains harboring insecticidal toxins. Only 5 strains were in clade 1, which has the highest concentration of mammalian pathogens (including *B. anthracis*, etiological agent of anthrax).

The per site nucleotide diversity (Π) [15], [16] of the 84 Rockville strains was almost identical to that of the greater population of 891 other isolates in the pubmlst.org database (both 0.04 substitutions per site). eBURST analysis [17] revealed that a number of the STs were members of clone groups that were unique to the sampling site or contained other *B. cereus* strains previously isolated from Maryland (**Figures S2 and S3**). Analysis with the UniFrac phylogeny-based comparison tool [18] showed that the community of isolates from Rockville was significantly different (P<0.01) to another collection of 219 *B. cereus* strains isolated from soil and leaves at a single site in the United Kingdom (using the same collection methods as this study [11]). However, certain STs from clade 2 (ST 111, 223 and 295) were represented by multiple strains in both the UK and Rockville strain sets. Thus the *B. cereus* population structure at each site appeared to consist of both cosmopolitan and endemic strains.

## Discussion

Microfluidics, a group of technologies based on the manipulation of microliter and nanoliter fluid volumes, emerged as a hybrid of molecular biology and microelectronics in the early 1990's [19]. Microfluidics devices make use of the high surface area to volume ratio of liquids in small channels, reservoirs, and chambers for rapid thermal transfer and transport and abbreviated reaction times. Small reaction volumes reduce the expense of reagents and require smaller instrumentation. The inherent ease of automation facilitates cost savings through reducing labor, cuts back on operator error, and allows the possibility of adapting instrumentation for forward deployment in the field. In this work, we detail the use of parallel technologies for very rapid microfluidic based thermal cycling and electrophoretic separation that significantly reduce labor costs for high-throughput PCR and DNA sequencing. The reaction vessels are plastic chips capable of large-scale manufacture by injection molding. The workflow can be improved further in the future by automating genomic DNA extraction and the transfer of samples between the sample preparation module, thermal cycler and electrophoretic separation module. These enhancements are currently in development.

MLST has been limited by its expense and inconvenience (to purchase, maintain and house several pieces of laboratory equipment and track data). mMLST could spur the emergence of new applications by providing a process flow as simple and inexpensive as placing bacterial cells in a well of a biochip, inserting the biochip in an instrument, and returning a few minutes later to read a set of nucleotide sequence profiles. Obviously, mMLST has many uses for high-throughput typing in the clinic. In regard to this arena, we have developed an mMLST typing scheme for *Chlamydia* (manuscript in preparation) and performed pilot studies on *Staphylococcus aureus* and *Streptoccus pneumoniae* (**Data S4** and **Text S2**). Another potential application for mMLST, previewed in this pilot study, is the extensive biogeographic surveillance of bacterial pathogen and near-neighbor populations to build up a profile of microvariation and demographic structure. MLST has a greater power for resolving subtypes than rRNA gene sequencing [20] and is one to two orders of magnitude less expensive than whole genome shotgun sequencing multiple individual strains. MLST-based studies have demonstrated the localized diversity of cosmopolitan bacterial species [21], [22]. Comprehensive sampling may allow opportunistic pathogens to be linked to specific geographic locales or environments by comparing the MLST diversity at the test site in similar panels from known locations using metrics such as UniFrac [18] or shared richness [23]. It may also be possible to build models of the geographical ranges of a number of clonal groups and estimate geolocation from overlapping the different STs found in the sample. This information would be particularly valuable for establishing forensic attribution of biothreat attacks.

## Materials and Methods

### Collection and Isolation of *B. cereus* Strains

Soil was collected from Rockville, Maryland (approximate sampling location: +39° 3′ 28.40″, −77° 6′ 54.90″) in September 2007. The basic method for bacterial isolation was adapted from Collier et al [11]. Soil was agitated with sterile sand in saline solution and spread over *B. cereus* selective agar plates (Oxoid Limited, Cambridge, United Kingdom). After overnight incubation at 30°C, pale blue colonies were picked and purified by re-streaking. Four mycoid colony morphology strains (approximately in proportion to the number of small round colonies) were also selected. Colonies were resuspended directly in 10 mM TE (pH 8.0) prior to DNA isolation.

### *B. cereus* DNA Purification

Approximately 40 µL of pelleted *B. cereus* sample was utilized for a guanidinium/silica binding-based purification [24] of total genomic DNA. The bacterial pellet was added to a tube containing 170 µL of lysis solution. The tube was vortexed for 10 seconds, briefly centrifuged, and incubated at 56°C with agitation for 15–30 minutes. 80 µL of absolute ethanol was added to the lysate, and the tube was vortexed and briefly centrifuged. Extract was transferred into a spin column (Quiagen, Valencia, CA) with a 7 mm diameter silica fiber filter for DNA binding. Lysate loading was performed by centrifugation at 6,000 g for 1 minute, and the column was washed twice. The filter was dried prior to elution in 30 µL TE (10 mM Tris-HCl, 1 mM EDTA; pH 8.0).

### Microfluidic PCR Amplification

Final concentrations of reaction components are: 1x Fast Buffer I and 0.225 U SpeedSTAR™ HS DNA Polymerase (Takara BIO, Madison, WI); 200 µM dNTPs; 250 nM primers and 0.5 ng template DNA. For *B. cereus* MLST amplification, the thermal cycling program began with a 70-second activation at 95°C followed by 30 cycles (4 seconds at 95°C, 10 seconds at 56°C and 10 seconds at 72°C). Total run time was approximately 17.5 minutes. The cycling profile is shown in **Figure 2**. A variety of enzymes are suitable for rapid amplification were tested [25]. Amplified products were manually retrieved from each reaction chamber and directly used as templates for Sanger sequencing. Conventional tube amplification was also performed on strains that did not generate PCR products for all loci.

### Microfluidic Sanger Sequencing

The BigDye® Terminator v3.1 cycle sequencing kit (Applied Biosystems, Foster City, CA) was adapted for implementation using the rapid thermal cycler and microfluidic PCR biochip described above. Each 7-plex PCR reaction was subjected to fourteen independent Sanger sequencing reactions (seven forward and seven reverse), with each reaction containing one sequencing primer; primers were identical to those utilized in PCR. Each reaction mix contained 1 µL DNA from the multiplex PCR reaction in a total volume of 7 µL. Thermal cycling begins with 60 seconds activation at 95°C, followed by 30 cycles (5 seconds at 95°C, 10 seconds at 50°C, and 40 seconds at 60°C). Total time per cycle was 32.9 seconds. Sequenced samples were manually retrieved from each reaction chamber and sample volume was brought to 10 µL with water for post-sequencing clean-up either by ethanol precipitation or ultrafiltration using a 30 kD Microcon/RC ultrafiltration YM type membrane (Millipore, Billerica, MA). Final volume of purified DNA was 13 µL for both methods.

### Microfluidic Separation and detection

The Genebench FX™ system was used to separate DNA based on fragment size by microfluidic biochip electrophoresis, and excitation and detection of labeled DNA fragments was accomplished by laser-induced fluorescence detection. Separation of the DNA fragments took place within a 16-sample microfluidic biochip that was prefilled with linear polyacrylamide sieving matrix. The 13 µl samples of cleaned-up sequencing reaction were placed into the sample reservoirs of the separation biochip and subjected to electrophoresis. Fragments arriving at the detection zone of the biochip were exposed to 488 nm laser excitation. Emitted fluorescence was collected by an optical train and transmitted to a color-specific detector. The electrophoretic separation took place in parallel for all 14 samples and detection process required approximately 30 minutes. Raw electropherograms generated on Genebench FX were transferred to a server for subsequent data processing and basecalling. Raw data electropherograms were basecalled using nnimbc4 (NNIM LLC, Salt Lake City, UT). Forward and reverse reads were aligned by importing the sequences in Codon Code Aligner (CodonCode Corporation, Dedham, MA) and incorporating a “standard” gene to truncate and align only the desired fragment lengths. DNA electropherograms and assembled allele loci were submitted to the curated *B. cereus* MLST web resource (www.pubmlst.org) as strain IDs 863–946.

### Phylogenetic analysis

MLST data was downloaded from the www.pubmlst.org website and analyzed using pubmlst online tools [26], DnaSP [27], MEGA4 [16], and UniFrac [18].

## Supporting Information

Figure S1 Phylogeny of *B. cereus glp* allele. Shows that many alleles from partially sequenced strains are from outgroup strains. Dark blue square are alleles that were found only in strains with incomplete STs in this study (ie one or more alleles missing). Forest green squares are *glp* alleles with a mixture of complete and incompletes STs, light green were associated with complete STs. The evolutionary history was inferred using the Neighbor-Joining method. The bootstrap consensus tree inferred from 1000 replicates is taken to represent the evolutionary history of the taxa analyzed. Branches corresponding to partitions reproduced in less than 50% bootstrap replicates are collapsed. The evolutionary distances were computed using the Maximum Composite Likelihood method and are in the units of the number of base substitutions per site. All positions containing gaps and missing data were eliminated from the dataset (Complete deletion option). There were a total of 372 positions in the final dataset.
(0.21 MB TIF)

Figure S2 E-BURST output clonal group containing only Rockville STs (Green type).
(2.35 MB TIF)

Figure S3 E-BURST groups containing both Rockville and diverse origin strains. E-BURST clonal group that is a mix of Rockville STs (green type), STs that are found both globally and in Rockville (Pink) and STs not found in Rockville.
(2.35 MB TIF)

Figure S4 Quality scores for all seven loci. Quality score plots for all seven MLST loci. Each plot was prepared in a similar manner to that described for figure 3. Plots represent the average of 180-200 electropherograms.
(1.56 MB TIF)

Data S1 Summary of pubmlst data submission. Each submitted isolate are broken down by alleles and Sequence Type.
(0.08 MB XLS)

Data S2 Breakdown of abundance by Sequence Type and Allele. Abundance charts for each data type.
(0.13 MB XLS)

Data S3 Aggregated quality score data. Columns are 1) position from start of trace, 2) numbers of electropherograms recording data 3) mean Q score 4) standard deviation.
(0.14 MB TXT)

Data S4 Zipped file containing 14 sequence trace files (.phd format) of sequencing runs described in supplemental text S2.
(0.22 MB ZIP)

Text S1 scf-qscores.pl perl script. Perl script used to generate data for figure 3.
(0.00 MB TXT)

Text S2 mMLST sequencing of *S. aureus* and *S. pneumoniae*.
(0.34 MB RTF)
