# Supplementary figures and images for: Rapid Multi-Locus Sequence Typing Using Microfluidic Biochips
Source: PLoS One. 2010 May 12;5(5):e10595. doi: 10.1371/journal.pone.0010595 (PMC2868872; doi:10.1371/journal.pone.0010595)

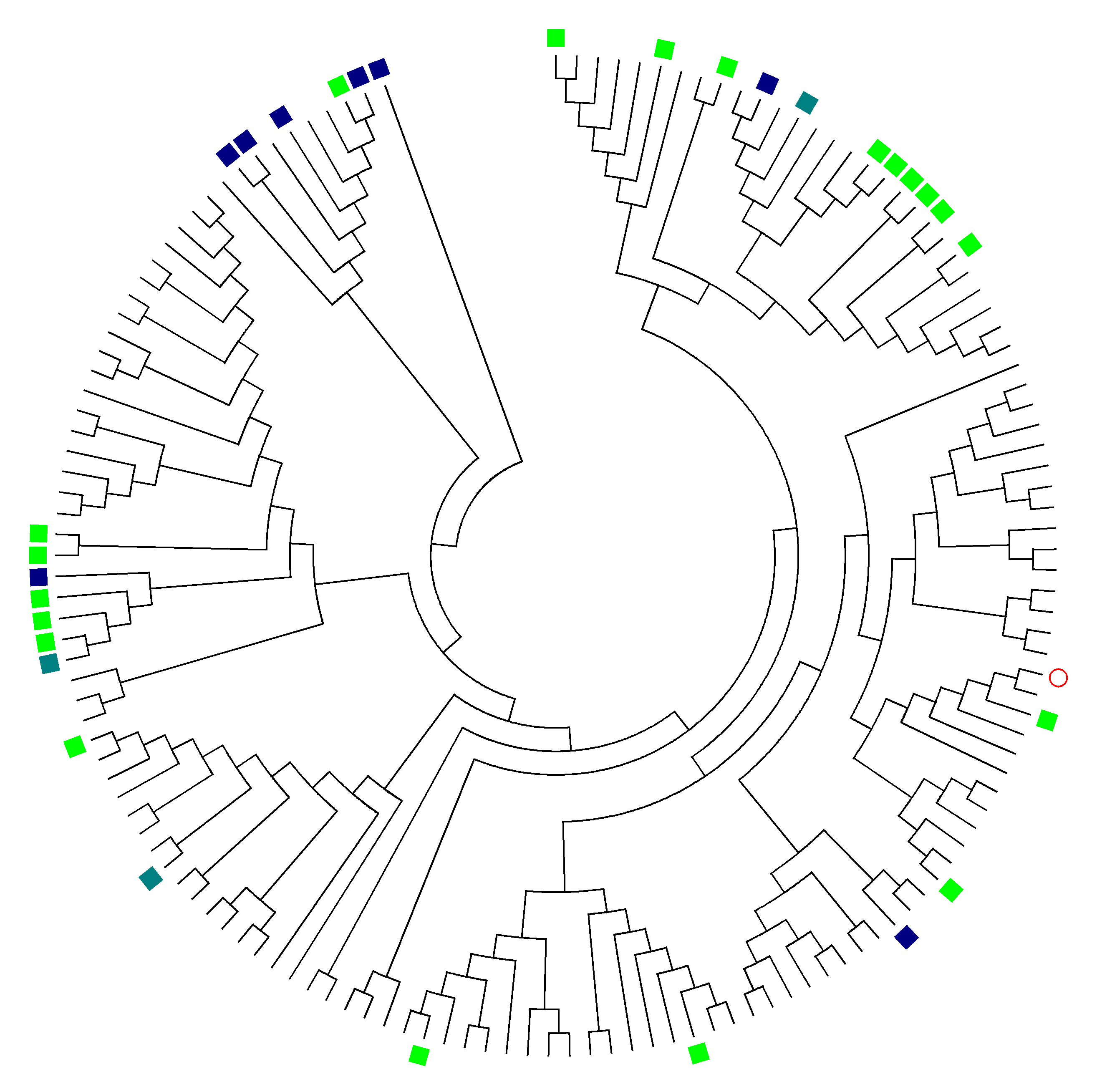

Supplement: Figure S1 — Phylogeny of B. cereus glp allele. Shows that many alleles from partially sequenced strains are from outgroup strains. Dark blue square are alleles that were found only in strains with incomplete STs in this study (ie one or more alleles missing). Forest green squares are glp alleles with a mixture of complete and incompletes STs, light green were associated with complete STs. The evolutionary history was inferred using the Neighbor-Joining method. The bootstrap consensus tree inferred from 1000 replicates is taken to represent the evolutionary history of the taxa analyzed. Branches corresponding to partitions reproduced in less than 50% bootstrap replicates are collapsed. The evolutionary distances were computed using the Maximum Composite Likelihood method and are in the units of the number of base substitutions per site. All positions containing gaps and missing data were eliminated from the dataset (Complete deletion option). There were a total of 372 positions in the final dataset. (0.21 MB TIF) [file pone.0010595.s001.tif]

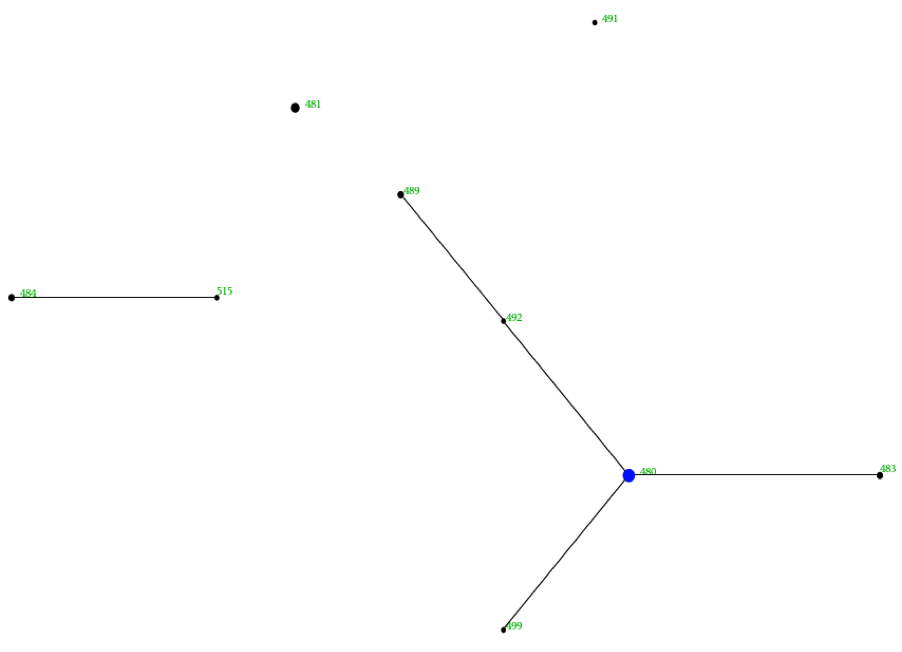

Supplement: Figure S2 — E-BURST output clonal group containing only Rockville STs (Green type). (2.35 MB TIF) [file pone.0010595.s002.tif]

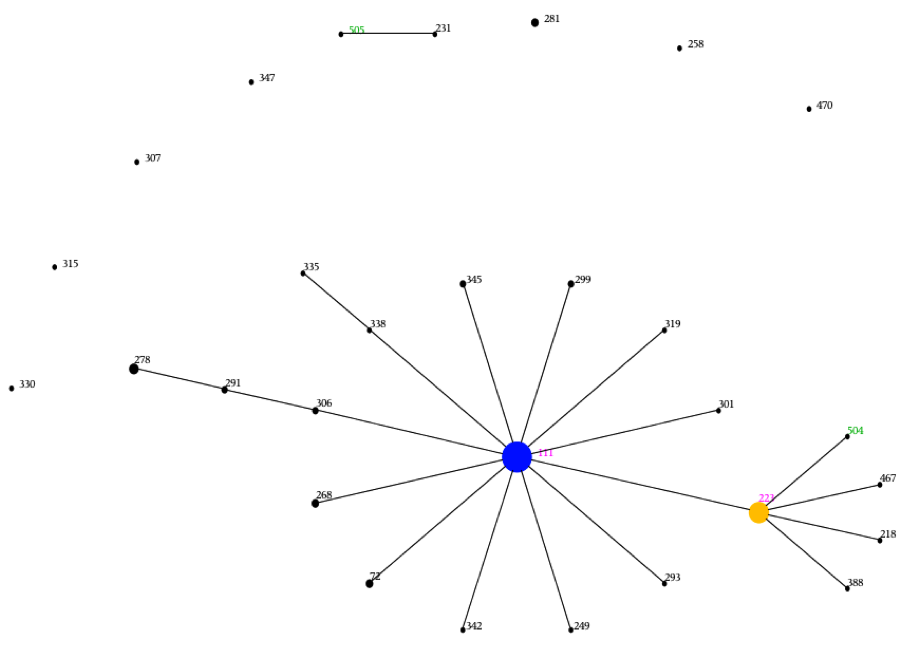

Supplement: Figure S3 — E-BURST groups containing both Rockville and diverse origin strains. E-BURST clonal group that is a mix of Rockville STs (green type), STs that are found both globally and in Rockville (Pink) and STs not found in Rockville. (2.35 MB TIF) [file pone.0010595.s003.tif]

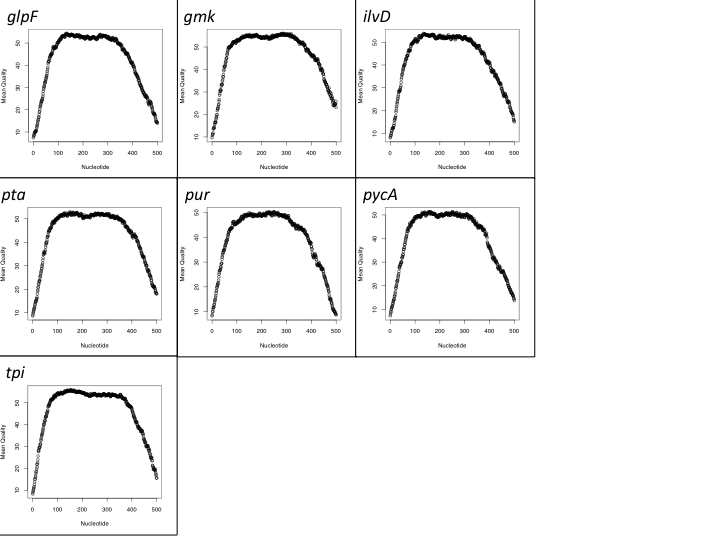

Supplement: Figure S4 — Quality scores for all seven loci. Quality score plots for all seven MLST loci. Each plot was prepared in a similar manner to that described for figure 3. Plots represent the average of 180-200 electropherograms. (1.56 MB TIF) [file pone.0010595.s004.tif]
